# Hospital detention of mothers and their infants at a large provincial hospital: a mixed-methods descriptive case study, Lubumbashi, Democratic Republic of the Congo

**DOI:** 10.1186/s12978-019-0777-7

**Published:** 2019-07-22

**Authors:** Karen D. Cowgill, Abel Mukengeshayi Ntambue

**Affiliations:** 1 School of Interdisciplinary Arts and Sciences, University of Washington Tacoma, Tacoma, USA; 2 Department of Global Health, University of Washington, Seattle, USA; 3 School of Public Health, University of Lubumbashi, Lubumbashi, Democratic Republic of the Congo

**Keywords:** Human rights abuses, Maternal health services, Democratic Republic of the Congo, Health expenditures, Poverty, Delivery, Obstetric

## Abstract

**Background:**

The practice of detaining people who are unable to pay for health care services they have received is widespread in many parts of the world. We aimed to determine the proportion of women and their infants detained for inability to pay for services received at a provincial hospital in the Democratic Republic of the Congo during a 6-week period in 2016. A secondary objective was to determine clinical and administrative staff attitudes and practices about payment for services and detention.

**Methods:**

This mixed-methods descriptive case study included a cross-sectional survey and interviews with key informants.

**Results:**

Over half (52%) of the 85 women who were in the maternity ward at Sendwe Hospital and eligible for discharge between August 5 and September 15, 2016 were detained for 1 to 30 days for outstanding bills of United States dollars (USD) 21 to USD 515. Women who were detained were younger, poorer, and had more obstetric complications and caesarean sections than other women. In addition, over one quarter of the infants born to these women had died during delivery or in the first three days of life. Key informant interviews normalized detention as an unfortunate but inevitable consequence of patient poverty and health system resource constraints.

**Conclusions:**

Detention of women and their infants is common at this hospital in the DRC. This represents a violation of human rights and a systemic failure to ensure that all people have access to essential health services and that they not suffer financial hardship due to the price of those services.

## French abstract

**Contexte:**

La détention des personnes qui sont incapables de payer les soins de santé reçus est répandue dans plusieurs régions du monde. Nous avons voulu déterminer la proportion des femmes et leurs enfants détenus après accouchement dans un hôpital provincial en République Démocratique du Congo (RDC) à travers une période de six semaines en 2016. Un objectif secondaire était de déterminer les attitudes et pratiques du personnel clinique et administratif sur le paiement pour les actes et sur la détention.

**Méthodes:**

Cette étude de cas descriptive à méthodes mixtes a inclus une enquête transversale et des entrevues avec des informateurs clés parmi le personnel administratif et clinique de l’hôpital.

**Résultats:**

Plus de la moitié (52%) des 85 femmes à la maternité de l’Hôpital Sendwe qui étaient éligibles à sortir entre le 5 août et le 15 septembre 2016, ont été détenues pendant 1–30 jours pour des factures non-payées de 21 à 515 dollars américains. Les femmes détenues étaient plus jeunes, plus pauvres, et ont eu plus des complications que celles qui ne l’étaient pas. En plus, plus d’un quart des enfants de ces femmes sont morts pendant l’accouchement ou dans les trois premiers jours de vie. Les informateurs clés ont considéré la détention comme conséquence regrettable mais acceptable et inévitable à cause de la pauvreté des patientes et des ressources limitées du système de santé.

**Conclusions:**

La détention des femmes et de leurs enfants est fréquente dans cet hôpital en RDC. Cela représente une violation des droits humains et un échec systémique par rapport à l’assurance à l’accès aux services de sante essentielles pour tout le monde et l’évitement des difficultés financières dues aux prix de ces services.

## Spanish abstract

**Contexto:**

La práctica de detener personas incapaces de pagar para servicios de salud que han recibidos es corriente en muchas partes del mundo. Hemos querido determinar la proporción de mujeres y sus bebes detenidos por incapacidad de pagar por los servicios recibidos en un hospital provincial en la República Democrática del Congo durante un periodo de seis semanas en 2016. Un objetivo secundario fue de determinar las actitudes y prácticas del personal clínico y administrativo en cuanto al pagamiento para los servicios y a la detención.

**Métodos:**

Este estudio de caso descriptivo de métodos mixtos comprendió una encuesta transversal y entrevistas de informantes claves.

**Resultados:**

Más de la mitad (52%) de las 85 mujeres en la unidad de maternidad del Hospital Sendwe que fueron elegibles para dejar el hospital entre el 5 de agosto y el 15 de septiembre 2016 fueron detenidas entre 1 a 30 días para facturas no pagadas de entre 21 y 515 dólares estadounidenses. Mujeres detenidas eran más joven, más pobre, y tuvieron más complicaciones obstétricas y cesáreas que las demás mujeres. Además, más de un cuarto de los bebes de estas mujeres habían muerto durante el parto o en los primeros tres días de vida. Las entrevistas de los informantes claves han normalizado la detención como consecuencia desafortunada pero inevitable de la pobreza de las pacientes y de las carencias del sistema de salud.

**Conclusiones:**

La detención de mujeres y de sus bebes es corriente en este hospital de la RDC. Lo mismo representa una violación de los derechos humanos y un fallo sistémico de asegurar que toda persona tenga acceso a los servicios de salud esenciales y que no sufra dificultades finánciales debido al precio de estos servicios.

## Plain English summary

The practice of detaining people who are unable to pay for health care services they have received is widespread in many parts of the world, although it is a violation of human rights. We aimed to document post-partum detention of mothers and their infants at a provincial hospital in the Democratic Republic of the Congo (DRC), where about 90% of people pay out of pocket for health care. This study included a survey and interviews with hospital administrators and clinical staff in 2016. We found that over half of the 85 women who were in the maternity ward at Sendwe Hospital and eligible for discharge between August 5 and September 15, 2016 were detained for 1–30 days for outstanding bills that ranged from $21–$515. Women who were detained were younger, poorer, and had more complications and caesarean sections with this birth than other women. Hospital administrators and clinical staff considered detention to be unfortunate but a normal and inevitable consequence of patient poverty and health system resource constraints. Detention of women and their infants is common at this hospital in the DRC. The Global Strategy for Women’s, Children’s and Adolescents’ Health [1] provides a roadmap to address the complex financial, human rights, and quality-of-care issues raised by this study.

## Background

Forced detention in hospitals -- the practice of detaining people who are unable to pay for health care services they have received -- is widespread in many parts of the world. News media, nongovernmental organizations, and researchers have reported on it in Africa, Asia, and Latin America [2, 3]. Cases in which post-partum women and their infants have been detained are among those commonly reported, although the practice is not limited to maternity units [4–8].

Hospital detention is an open secret: not only have news media and filmmakers in several countries reported on it (e.g., Kenya [6, 9–14], Ghana [15–17], Zimbabwe [18, 19], Nigeria [20–22], Uganda [5, 23], DRC [24–27], Cameroon [8], India [28, 29], Indonesia [30]), but also politicians have taken advantage of it to ingratiate themselves with voters by paying individuals’ bills to free them [6, 24]. African- and international-based human rights groups have campaigned to raise awareness of the practice, notably in Burundi [31], Kenya [32], and the Democratic Republic of the Congo (DRC) [33]. Some governments have denounced the practice (Zimbabwe; [19]) or legislated against it (Philippines; [34]), but it continues despite official efforts.

Detention in health care facilities is one of seven categories of disrespect and abuse that women encounter in facility-based childbirth, as set out by Bowser and Hill in their seminal 2010 landscape analysis [35]. A grassroots organization, the White Ribbon Alliance [36], promulgated a Respectful Maternity Care charter that details rights that correspond to each of Bowser and Hill’s categories. In the case of detention, the corresponding right is to “liberty, autonomy, self-determination, and freedom from coercion.” [37] This right is upheld by several international standards, including the Declaration of the Elimination of Violence Against Women (DEVAW), 1994, Article 1; the International Covenant on Economic, Social and Cultural Rights (ICESCR), 1976, Article 1; the International Planned Parenthood Federation Charter on Sexual and Reproductive Rights, 1996, Article 2; the International Covenant on Civil and Political Rights (ICCPR), 1966, Article 9.1, 18.2; and the Universal Declaration on Bioethics and Human Rights, Article 5 [37]. In addition, Article 37(b) of the Convention on the Rights of the Child states that “No child shall be deprived of his or her liberty unlawfully or arbitrarily.” [38] The World Health Organization (WHO) cited the Bowser and Hill report in its 2014 statement, *The prevention and elimination of disrespect and abuse during facility-based childbirth*, and called for research “to better define, measure and understand disrespectful and abusive treatment of women during childbirth, and how it can be prevented and eliminated.” Bohren et al. [39] created an evidence-based typology in which they defined and expanded on the categories of disrespect and abuse laid out by Bowser and Hill. In this typology, detention in facilities is a manifestation of “loss of autonomy” under the umbrella theme of “poor rapport between women and providers.” Only three studies with data on detention – one quantitative and two qualitative – met the criteria for inclusion in their systematic review [39], underscoring the paucity of data on this topic.

In February 2015, we found women on the maternity ward at the Jason Sendwe Provincial Hospital in Lubumbashi, DRC, the public referral hospital for the province of Katanga, who were detained for non-payment of fees. A subsequent retrospective review of medical records for the years 2014–15 showed that some women and their infants had very long stays in the maternity unit, in one case for 73 days. However, documentation in medical records was sparse and we could not retrospectively establish the reasons for the protracted lengths of stay.

To better understand this phenomenon, we designed a mixed-methods descriptive case study of post-partum women and their infants at Sendwe Hospital. Our primary objectives were to determine the proportion of women detained for inability to pay for services received and to describe the trajectory of care, obstetric history, expenditures, and length of stay of women in the maternity unit during a 6-week period in 2016. A secondary objective was to determine clinical and administrative staff attitudes and practices about payment for services and detention.

## Methods

To address our primary objectives, we conducted a cross-sectional survey. We approached women who were in the maternity unit following delivery between August 5 and September 15, 2016. With their consent, we administered a questionnaire with closed questions about demographic and socioeconomic characteristics, the course and outcome of the pregnancy of which they had recently been delivered, and their care-seeking trajectory (i.e., where they initially presented for care, whether they were referred, how they reached the health care facility) and expenditures for the delivery. We followed up with nursing staff to determine which patients had been medically cleared for discharge and of those, which had been discharged by the expected day, which is normally three days after an uncomplicated vaginal delivery, or ten days after a caesarean section. We considered a woman to be detained if she had been medically cleared for discharge but denied the “bon de sortie,” or discharge papers, because she had not paid in full for care received. To leave the hospital premises, patients must show the bon de sortie and have it signed by guards on their units and at the hospital’s main gate. We tabulated descriptive statistics (proportions of categorical variables and means and medians of continuous variables) by detention status. Since the study was not designed to test any hypotheses, we did not report tests of statistical significance.

The second component of our study consisted of interviews of hospital staff using a semi-structured interview guide about the official and actual fees for delivery at the hospital, their understanding of why women were detained at the hospital, and their opinions about the importance of the situation and what should be done with women who haven’t paid for their delivery care. Three of the clinical staff comprised a focus group, while the others were interviewed individually. All ten hospital staff refused recording, so the interviewer (AMN) wrote detailed notes during and immediately following the interviews. We did not perform a formal content analysis since we did not have verbatim transcriptions of the interviews, but we summarized and synthesized information from the interviews and related them to the themes put forth in the typology by Bohren et al. [39].

## Results

### Cross-sectional study

Over half of the 85 women who were in the maternity ward at Jason Sendwe Hospital and eligible for discharge between August 5 and September 15, 2016 were detained – i.e., they were not issued discharge papers on the expected day because they had not paid some or all of the fees incurred from their or their infant’s care (Table 1).

**Table 1:** Sociodemographic characteristics of women who delivered at Jason Sendwe Hospital, Lubumbashi, Democratic Republic of the Congo

	Cross-Sectional Study Aug-Sep 2016	
	Not Detained (*n* = 41)	Detained (*n* = 44)
Age mean (sd) [median, min-max]	29.0 (7.7) [17–42]	25.7 (6.7) [16–42]
Highest level of education		
None	1 (2.4%)	2 (4.5%)
Some primary	1 (2.4%)	2 (4.5%)
Completed primary	1 (2.4%)	1 (2.3%)
Some secondary	20 (48.8%)	24 (54.5%)
Completed secondary	13 (31.7%)	12 (27.3%)
Post-secondary	5 (12.2%)	3 (6.8%)
Occupation		
Homemaking	28 (68.3%)	25 (56.8%)
Selling	4 (9.8%)	6 (13.6%)
Farming	0	0
Civil service	1 (2.4%)	0
Private company	0	3 (6.8%)
Trades	6 (14.6%)	7 (15.9%)
Student	0	2 (4.6%)
Other	2 (4.9%)	0
Marital status		
Married – monogamous	40 (97.6%)	35 (80.0%)
Married – polygamous	1 (2.4%)	1 (2.3%)
Divorced	0	0
Widowed	0	2 (4.6%)
Single	0	6 (13.6%)
Age of partner (years): mean (sd) [median, min-max]	*n* = 37:36.4 (8.1) [22–55]	*n* = 35:34.5 (8.1) [22–50]
Age difference (years): mean (sd) [median, min-max]	7.1 (4.1) [6, −1-20]	7.6 (3.7) [2–16]
Partner’s highest level of education	*n* = 41	*n* = 38
Unknown	1 (2.4%)	1 (2.6%)
None	1 (2.4%)	1 (2.6%)
Some primary	0	0
Completed primary	0	0
Some secondary	6 (14.6%)	7 (18.4%)
Completed secondary	19 (46.3%)	10 (26.3%)
Post-secondary	14 (34.1%)	19 (50.0%)
Partner’s occupation	*n* = 41	*n* = 38
Homemaking	0	1 (2.6%)
Selling	3 (7.3%)	4 (10.5%)
Farming	0	1 (2.6%)
Public functionary	2 (4.9%)	2 (5.3%)
Public company	2 (4.9%)	2 (5.3%)
Private company	13 (31.7%)	4 (10.5%)
Trades	15 (36.6%)	21 (55.3%)
Student	0	0
Other	6 (14.6%)	3 (7.9%)
Respondent’s relationship to head of household		
Partner	38 (92.7%)	34 (77.3%)
Sister	0	1 (2.3%)
Daughter	1 (2.4%)	3 (6.8%)
Self	0	1 (2.3%)
Daughter-in-law	1 (2.4%)	4 (9.1%)
Other	1 (2.4%)	1 (2.3%)
SES		
Home ownership		
Renter	28 (68.3%)	31 (70.5%)
Own/family home	13 (31.7%)	11 (25.0%)
Other	0	2 (4.6%)
SES Index*		
Below median (< 5)	12 (29.3%)	25 (56.8%)
Median [6]	17 (41.5%)	13 (29.6%)
Above median (> 5)	12 (29.3%)	6 (13.6%)
Number of people in HH mean (sd) [median; min-max]	6.1 (3.8) [5; 1–18]	5.1 (2.5) [5; 2–11]
Upper limit of HH daily income/person (USD**) Mean (sd) [median; min-max]	*n* = 37 $1.78 (1.24) [$1.50; 0.28–6.06]	*n* = 39 $2.01 (2.12) [$0.87; 0.28–9.09]

* SES Index based on reported wall and roof construction, water, lighting, fuel source, type of toilet, goods including electronics, appliances, fields

** USD: United States dollars (calculated using historical exchange rate between Congolese francs (CDF) and USD on August 30, 2016, midway through the study (990 CDF/USD; http://www.xe.com/currencytables/?from=CDF&date=2016-08-30 accessed 30 Aug 2017)

In the sample overall, the mean upper limit of the self-reported household monthly income range per household member was equivalent to United States dollars (USD) 57, or USD 1.90/person/day, with a median of USD 1.21/person/day and a range from USD 0.28/person/day to USD 9.09/person/day. Women who were detained had less wealth as measured in land, housing materials, and household goods, and had a lower median daily income per household member, although their mean income was higher due to a couple of outliers. Eight of the ten women who were detained for more than a week had per-person, per-day household incomes less than USD 1.90.

Detained women were on average more than 3 years younger than those not detained and had had about one fewer pregnancy (these observations were correlated; r = 0.73 (*p* < 0.0001)). A lower proportion of women who had been detained were in a current partnership: all the women who were discharged on time were in current partnerships, but close to 20% of those detained were either widowed or never married. Educational attainment and occupation of the two groups of women was similar, as was their partners’ education; however, far fewer of the partners of detained women worked in private companies, and more worked in the trades.

Table 2 shows that more women who were detained for non-payment had obstetric complications and caesarean sections than women who were discharged on the expected date. A higher proportion of detained than non-detained women had delivered by caesarean section in the past. At the delivery in the study period, women who were detained more often had complications, and a greater number of complications, including caesarean section, than women who were not detained (mean number of complications per woman 1.2 vs. 0.7). Obstructed labor and eclampsia were particularly frequent, as were premature rupture of membranes and antepartum hemorrhage. More detained women had twins. No maternal deaths were reported during the study period, but 22 of the 85 (26%) women interviewed lost the infant delivered during this hospital stay on or before the third day of life: 14 (34%) of the 44 women who were detained, and 8 (20%) of the 41 women who were not detained. Of the 91 infants delivered (6/85 women delivered twins), 7 (8%) were stillborn (5 fetuses died before and 2 during delivery per the mothers’ report). Of the 84 infants born live, 15 (18%) had died by the time of interview: 10 on the first day of life, 4 on the second day, and 1 on the third day. Prematurity was reported to be the cause of death in 4 cases; 2 died of “breathing problems,” 5 of other causes, and 4 of unknown cause.

**Table 2:** Obstetric history of women who delivered at Jason Sendwe Hospital, Lubumbashi, Democratic Republic of the Congo

	Cross-Sectional Study Aug-Sep 2016	
	Not Detained (*n* = 41)	Detained (*n* = 44)
Number of pregnancies mean (sd) [median; min-max]	4.2 (3.4) [3; 1–13]	3.4 (2.6) [2.5; 1–12]
Number of deliveries mean (sd) [median; min-max]	3.9 (3.3) [3; 1–13]	2.9 (2.1) [2; 1–8]
Number of Cesarean sections		
1	11 (26.8%)	22 (50.0%)
2	1 (2.4%)	3 (6.8%)
3	1 (2.4%)	0
Attended prenatal care at least once during this pregnancy	38 (92.7%)	41 (93.2%)
< 4 prenatal care visits	14 (34.1%)	17 (38.6%)
4 prenatal care visits	11 (26.8%)	13 (29.5%)
> 4 prenatal care visits	13 (31.7%)	11 (25.0%)
Complications this delivery (any)	22 (53.7%)	31 (70.5%)
1 complication	16 (39.0%)	16 (36.4%)
2 complications	4 (9.8%)	9 (20.5%)
3 complications	2 (4.9%)	5 (11.4%)
4 complications	0	0
5 complications	0	1 (2.3%)
Type of complication		
PROM (premature rupture of membranes)	6 (14.6%)	7 (15.9%)
Antepartum hemorrhage	4 (9.8%)	7 (15.9%)
Obstructed labor	10 (24.4%)	22 (50.0%)
Post-partum hemorrhage	3 (7.3%)	2 (4.6%)
Retained placenta	2 (4.9%)	3 (6.8%)
Uterine rupture	1 (2.4%)	1 (2.3%)
Eclampsia	2 (4.9%)	9 (20.5%)
Placental abruption	0	0
Fetal death	2 (4.9%)	3 (6.8%)
Early neonatal mortality	6 (14.6%)	11 (25.0%)
Mode of delivery		
Vaginal, without instruments	30 (73.2%)	18 (40.9%)
Cesarean	10 (24.4%)	26 (59.1%)
Vaginal, with instruments	1 (2.4%)	0
Birth weight (grams)		
Singletons mean (sd) [median; min-max]	*n* = 33 3153 (526) [3200; 1200–3960]	*n* = 36 2985 (767) [2950; 1300–4500]
Twins mean (sd)	1 set 2750	5 sets 2288 (323)
Preterm birth (< 37 weeks self-reported gestational age)	10 (24.4%)	16 (36.4%)

Table 3 shows that more women who were eventually detained at Sendwe had sought care initially at a health center, and they paid more at the first facility before being referred to Sendwe than those who were not detained. Three of the 85 women (3.5%) in the sample overall reported that they had intended to deliver at home; forty women (47.0%) first sought care at a health center, the level of the health care system designated for uncomplicated deliveries. When transferred, they paid transportation costs.

**Table 3:** Trajectory of care and payment history of women who delivered at Jason Sendwe Hospital, Lubumbashi, Democratic Republic of the Congo

	Cross-Sectional Study Aug-Sep 2016	
	Not Detained (*n* = 41)	Detained (*n* = 44)
Trajectory: initial location of delivery		
Sendwe	24 (58.5%)	17 (38.6%)
Health Center	14 (34.1%)	26 (59.1%)
Home	2 (4.9%)	1 (2.3%)
Other	1 (2.4%)	0
Amount paid to initial health center in USD*, mean (sd) [median; min-max]	*n* = 15 11.92 (20.21) [2.02; 0–60.60]	*n* = 23 14.67 (22.21) [5.05; 0–90.91]
Mode of transport to hospital		
On foot	0	1 (2.3%)
Public transport	0	4 (9.1%)
Shared taxi	10 (24.4%)	5 (11.4%)
Private taxi	24 (58.5%)	30 (68.2%)
Private vehicle	7 (17.1%)	4 (9.1%)
Cost of transport to hospital in USD, mean (sd) [median; min-max]	*n* = 34 3.60 (3.46) [3.03; 0.20–15.15]	*n* = 39 3.88 (3.04) [3.54; 0.20–14.14]
Amount paid to Sendwe in USD, mean (sd) [median; min-max]	126.85 (145.72) CDF [42.42; 25.25–474.75]	262.45 (161.02) CDF [322.22; 23.23–620.20]
Amount remaining to pay in USD, mean (sd) [median; min-max]	NA	210.60 (132.71) [192.42; 21.31–515.15]
Length of stay (days); mean (sd) [median; min-max]	5.7 (3.7) [4; 2–16]	16.5 (10.7) [14.5; 4–43]
Extra days billed	NA	27 (61.4%)
Duration of detention (days); mean (sd) [median; min-max]	NA	7.5 (8.0) [5; 1–30]

*USD: United States dollars: For the retrospective study, we converted Congolese francs to US dollars using the historical exchange rate as of Jan 1, 2015 (923 CDF/USD http://www.xe.com/currencytables/?from=CDF&date=2015-01-01 accessed 29 Sept 2017), and for the cross-sectional study, we used the rate on August 30, 2016, midway through the study (990 CDF/USD; http://www.xe.com/currencytables/?from=CDF&date=2016-08-30 accessed 30 Aug 2017)

**Fig. 1:**
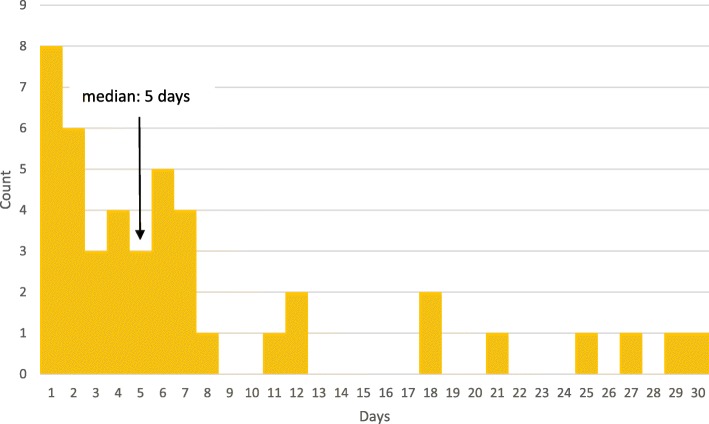
Duration of detention at time of survey of women and infants delivered at Jason Sendwe Hospital, Lubumbashi, Democratic Republic of the Congo, 2016

The detained women had already paid between USD 23 and USD 620 to Sendwe. In the sample overall, the mean amount women still needed to pay to obtain a discharge order was equivalent to USD 197 (standard deviation (sd) USD 167). Women who were detained owed more and had between USD 21 and USD 515 left to pay. Charges for the extra days due to detention were billed to 27/44 (61.4%) of women detained (Fig 1).

The figure shows that the median duration of detention at the time of the study was 5 days, with a maximum of 30 days; thirty-three women were detained for one week or less.

### Key informant interviews

To determine clinical and administrative staff attitudes and practices about payment for services and detention, one of us (AMN) conducted interviews with four supervisory/administrative staff who had all been appointed to their roles in 2011. We also interviewed six clinical staff, whose tenure ranged from 2.5 to 20 years, and three staff members who did not report the duration of their employment. Unless otherwise noted, at least two key informants independently provided the information summarized below. Where noted, subheadings refer to themes in the “Typology of the mistreatment of women during childbirth” [39].

#### Health system conditions and constraints – lack of resources [39]

Hospital administrators said the national Ministry of Public Health had paid only a fraction of the USD 15,000/month slated to support the hospital’s operating costs. They said that in all of 2015, only 3 monthly payments came from the government, and that by September 2016, when this study was conducted, only 3 monthly payments had come in 2016. They hoped the governor (a multi-millionaire businessman [40]) might subsidize the hospital’s costs.

#### Health system conditions and constraints – facility culture – unclear fee structures [39]

Key informants said that it was the former governor of (Haut-)Katanga^1^ Province who had set the price of an uncomplicated delivery at Sendwe Hospital at 19,000 CDF (USD 19) without consulting hospital administration. They said this price was disseminated on the news and other media, so women went to Sendwe expecting to pay this amount, which was less than the going rate elsewhere. However, they revealed that there is great variability in what fees are actually charged. There was consensus that the official price of an uncomplicated delivery was 19,000 CDF (USD 19), but that actual fees once supplies and medications were tallied rose to at least 37,000 CDF (USD 37). Further, the base price of a caesarean section was 302,700 CDF (USD 306), with additional fees assessed for the operating kit, medications (1500–55,000 CDF, or USD 1.50 to 56.00), and the obligatory 7-day neonatology unit stay of an infant born by caesarean section (70,000 CDF, or USD 71). The hospital itself set the price of a caesarean section based on its costs. Some acknowledged that caesarean sections were too expensive given the standard of living of families in Lubumbashi; one provider said, “even I wouldn’t be able to pay.”

#### Health system conditions and constraints - lack of resources - physical condition of facilities/staffing constraints/supply constraints [39]

Interviewees discussed effects of non-payment and detention for non-payment, saying that it prevents the hospital from functioning because beds are filled with women who can’t pay, and nurses can’t do their jobs properly. Nurses also sell disposable diapers and medications to offset their low salaries, which are low in part because some patients don’t pay.

#### Health system conditions and constraints – lack of policies [39]

All stated that there is no official policy in place regarding detention of patients for non-payment of fees. Interviewees often took a pragmatic approach to the problem of detention. Compromises mentioned included encouraging women to pay in installments; accepting half the fee or, “whatever she has”; and health care providers’ taking up a collection among themselves to buy food for detainees or to pay to release them. A nurse on the maternity unit said they bill the mother for only the first 3 days on the maternity unit if her baby is in the neonatology unit for 7 days, to avoid double-billing. One interviewee said that “known orphans, soldiers, or state employees” receive a 20% discount and cannot be detained.

Several talked about advocating to the administration for release of detained women. For example, one said that if a woman no longer had food, the staff might release her. Another said that after two months of detention, the administration would investigate to find out if a woman was really unable to pay. How the administration would determine this was unclear, and statements about whether the woman would be released if she was found unable to pay were inconsistent. Some interviewees described putting pressure on women who were detained to get them to pay. One reported tactic was to put two women in one bed. One administrator claimed that, “sometimes we intimidate them by taking the baby, under the pretext that we’ll keep the baby if they don’t pay,” however, this claim was not supported by statements from clinical staff.

Key informants said some women escape without paying: according to one interviewee, especially women whose babies have died, and according to another, at least one woman would escape per week. One interviewee proposed having some form of identification for women to reduce the chance that they would escape.

#### Failure to meet professional standards of care – neglect and abandonment – neglect, abandonment, or long delays [39]

Referral patterns also came up as an explanation for why women who were unable to pay ended up at Sendwe. Because Sendwe is the only fully public tertiary hospital in the province, it is a place to which other facilities, even the parastatal tertiary hospitals, refer insolvent women. One provider who used to work at one of the public general hospitals confirmed that they would write “transferred for lack of means” on the referral form of such women and send them to Sendwe. This meant that these women were essentially abandoned by providers and then subject to delays while they sought care elsewhere.

#### Why women are unable to pay

All interviewees invoked poverty and complications at delivery that increased charges as the primary reasons women were unable to pay. They agreed that women were rarely, if ever, unable to pay following an uncomplicated vaginal birth, and mentioned caesarean sections, which are expensive, as a common experience of women who were unable to pay; one interviewee said that women with eclampsia were those who paid the most. They pointed out that a woman rarely pays the fees alone; often the extended family takes up a collection among its members to pay her bill.

Reasons given for why women might be poor and unable to mobilize resources included abandonment by their families or partners, being young (implying unmarried), or having been impregnated (implying as a result of abuse or rape). One interviewee mentioned that some women who were unable to pay and hence detained had been displaced by conflicts elsewhere in the DRC.

All interviewees said that detention of women in the maternity ward was a major, serious problem. Many seemed to view these women with compassion, referring to them as destitute, but one said that non-payment was an expression of “ingratitude for the work we do.”

#### Verbal abuse – harsh language – judgmental or accusatory comments [39]

Some interviewees blamed women for their own detention, saying that because they hadn’t gone to prenatal care, they hadn’t planned for delivery, and weren’t aware of or hadn’t set aside money to pay the fees. Some said women were acting in bad faith and did not intend to pay, believing the state or a benefactor would pay their bill. If a woman was believed to be acting in bad faith, i.e., she could pay, but chose not to, providers would not advocate on her behalf. A couple of interviewees described women as being “used to it,” having been detained after previous deliveries and liberated by a benefactor. One interviewee averred that, “some women feel better when they stay here” because they have a bed and their family brings them food, suggesting that hospital detention provided a respite from duties at home.

#### What should be done

Interviewees proposed many alternatives that they believed might alleviate the problem of post-partum detention at Sendwe by making it easier for women to pay the fees or by changing the financing structure of the health care system. These included having women pay installments or deposits toward the price of the delivery during their pregnancies, establishing risk pools or a social insurance fund, increasing provider salaries, and providing state subsidies or changing government policy. Some suggested finding another facility or nongovernmental organization that would take women in. Others suggested relying on benefactors such as the first lady of the DRC, the former governor, Catholics who paid detained women’s bills as acts of charity during Lent, the church, or businesses such as Tigo, a mobile phone company.

## Discussion

This study shines light on forced hospital detention for non-payment of fees, one aspect of disrespectful and abusive care experienced by women who deliver in facilities. We documented the length of stay of 85 women who delivered at Sendwe Hospital in Haut-Katanga province in the DRC over a 6-week period in 2016 and found that 52% were not issued discharge papers on their expected day of release because they had not paid some or all of the charges incurred during their hospital stays. Outstanding fees ranged from USD 21 to 515, and women were detained for up to one month.

In other settings, researchers have reported on the phenomenon of detention, but few reports exist in the peer-reviewed literature that document its frequency and extent. A notable exception is the study by Okafor et al. [41], who found that, among 446 women interviewed at an infant vaccination clinic in Enugu, Nigeria, 98 (22%) reported that they had been detained in a facility following their infant’s delivery. Also, Mostert et al. [4] found 19 of 36 (53%) uninsured pediatric cancer patients in Uganda were detained for non-payment, with length of detention ranging from 2 to 21 days and averaging about a week. Other studies reporting the incidence of detention include one by Sando et al. [42], who found that, of post-partum women in a convenience sample recruited over a 6-month period at a hospital in Tanzania, 3/1954 (0.2%) had been detained. Another study by Asefa et al. [43] found one of 173 (0.6%) of a sample of women who delivered at four hospitals in Addis Ababa in 2013 had been detained. Drawing comparisons among these studies is complicated because of the great variation in the way authors defined study populations and selected samples, as well as in the underlying contexts and health-care systems in which the studies were conducted.

Some of the most comprehensive reports on detention are in the grey literature. In 2006 in Burundi, Human Rights Watch and the Association for the Promotion of Human Rights and Detained Persons documented extensive routine detention of all types of patients for non-payment, reporting that they found detained patients at nine of eleven hospitals [31, 44, 45]. A December 2017 review by the Centre on Global Health Security, an independent policy institute, provides a detailed overview of the issue of hospital detention for non-payment of fees [2]. There is agreement that the practice of hospital detention exists within and arises from a context of unequal resource distribution, inadequate funding of health care systems, and poor governance [46].

The income that women in our sample reported aligned with the 2016 United Nations Development Program Human Development Index, which estimated that 77% of DRC’s population lives on less than USD 1.90/day [47]. It is not surprising that those who were detained were the poorest and those with the highest fees, occasioned by complications during delivery. The average amount that women in our sample owed to Sendwe was equivalent to nearly 3.5 months’ income at USD 1.90/day, and some owed amounts equivalent to 9 months’ income. To add insult to injury, more than half of women who were detained for non-payment were charged for the additional days they were hospitalized, and all incurred opportunity costs since they were unable to work while detained.

The duration of detention was less than a week for most of the detained women in this sample. We suspect that our finding that thirty days was the longest any woman in our sample was detained at the time of our study implies that a benefactor paid to release detained women in the month before, as occurs periodically in this and other settings [6, 24]. However, since this was a cross-sectional study, we could not predict or observe how long these women would be detained before settling their debt or otherwise managing to leave the hospital. It is not uncommon for women to be detained longer at Sendwe. Study team members who visited the unit reported some women in our sample were still detained as of March 2017, six months after the end of the study. These women said that their infants had not received routine childhood vaccinations, meaning they were at increased risk of vaccine-preventable disease as well as nosocomial infection [48–50] and social and developmental deficits.

Women in our sample who initially sought care at health centers were effectively penalized if they developed complications. They forfeited any payments they had already made, had to pay for transportation from the facility where they initially presented to Sendwe, and then had to pay fees to Sendwe. They may also have faced delays in receiving appropriate care. The health care landscape in Lubumbashi is poorly regulated and difficult to navigate: in 2011, we identified 180 facilities providing maternity care in this city of 2.2 million [51]. The confusion of services between the lower and upper levels, and the large number and lack of regulation of private facilities, has created a system in which there is open competition between levels of care [52], and in which lower-level facilities may be reluctant to forgo income by referring patients elsewhere [53]. Thus, many women opt to attend a lower-level public facility or a private facility in hopes of receiving care of higher quality and/or lower price. If they develop a complication, they may end up at Sendwe, unable to pay the fees for their care.

A surprising finding of our study was the high proportion of fetal and neonatal mortality among infants born during the study period. Neonatal deaths had reportedly decreased after an international NGO provided a training course in 2015 which was attended by providers from Sendwe and the surrounding health zones of Lubumbashi. This supposedly reduced infant deaths at Sendwe Hospital by 40% [54]. However, we found a high proportion of infant mortality and stillbirth among women in our sample: one quarter had lost the infant they delivered during this hospital stay on or before the third day of life. This suggests that there are serious challenges for DRC’s meeting the Sustainable Development Goal of ending preventable deaths of newborns and reducing neonatal mortality to no more than 12 per 1,000 live births by 2030 [55].

The information from our key-informant interviews puts the findings of our cross-sectional study into context by revealing attitudes and practices of hospital staff and suggests factors that drive the practice of detention in this setting.

At the highest level are constraints on the hospital imposed by the failure of the DRC government to make monthly payments to support the hospital’s operating costs. Since the 1990s, the DRC has been characterized by extremely weak government structures -- it has been called by some a failed state [56], a non-state [57], or a violent kleptocracy [58]. The state progressively disengaged from financing the health care system and shifted the costs of health care to users [59], but without providing for insurance schemes that would distribute the payment burden [60, 61] or paying health care workers wages that would compete with the private sector [62].

The DRC has no national health insurance scheme; about 90% of households pay out of pocket for health care, and those out-of-pocket payments accounted for 43% of all health expenditures in 2015 [63]. Out-of-pocket payments, or user fees, make up the largest proportion of health care workers’ salaries [64, 65]. WHO has called user fees, “the most inequitable method for financing health care services.” [66] In fact, the fees charged at Sendwe Hospital for complicated deliveries are not aligned with what patients and their families can afford, as the quantitative data from our study show. Furthermore, the fee structure for delivery at Sendwe is unclear: women are charged for medications and supplies on top of provider services, and fees vary widely by type of complication.

On the one hand, fees are too high for patients to afford, and on the other hand, key informants aver that the revenue is too low to support the operating costs of the hospital and to pay hospital staff. Some staff resort to selling diapers and medications to generate income. Others have described the constraints faced by health care workers in the public sector in DRC, many of whom do not receive a formal salary and rely instead on a monthly “prime de risque,” or hazard pay, from the government [65, 67, 68].

One finding from the key-informant interviews was that there are no formal policies about imposing or suspending detention at Sendwe. There was consensus among key informants that the issue of detention for non-payment of fees constitutes a serious problem. They recognized it was poor treatment, but they normalized and accepted it as arising from systemic constraints in the face of which individual actors – administrators, staff, patients – were left to make ad hoc responses, apparently driven by judgments about whether women were deserving of help or not. Such judgmental attitudes may be passed on to providers during their training and perpetuated by facility culture [46, 69].

### Limitations

There are approximately 180 facilities that provide maternity care services in Lubumbashi [70]; this study gathered data from only one unit at one hospital in one six-week period. It is not possible to extrapolate from this study to estimate numbers detained at this facility or in the city of Lubumbashi, given the variation from one facility to another in whether patients are detained, as well as within facilities. Our effort to look at previous years’ data to get a longer view of detention was frustrated by the finding that most records did not include data on length of stay or reasons for stays that were longer than standard.

Other limitations of this study include the self-reported nature of some of the information, such as amounts owed or paid at this and other facilities where women might have sought care. Women who stated they were waiting for help or had no solution to pay their bills may have exaggerated their lack of options in the hope or expectation that study staff would pay their bills, despite the introductory informed consent statement that they would receive no gifts or incentives for their participation in the study interview. We collected only quantitative data from patients in response to a survey that posed closed questions; the survey did not capture qualitative data or details about the treatment women received while detained (e.g., denial of care or posting of guards). In another study, we have documented the high proportion of households in Lubumbashi that suffer catastrophic costs as a result of childbirth [71].

Population-based data that would document the incidence of detention would help make a case to implement and enforce policies, but – while it’s important to collect baseline data and monitor improvement – the priority is ending the practice. We surveyed households in Lubumbashi about hospital detention in a population-based survey in Lubumbashi conducted in May 2018 and expect that this will provide one of the first population-based estimates of the incidence of hospital detention for non-payment.

### Strengths

This study contributes to the literature on women’s experiences during childbirth and highlights a common but understudied aspect of disrespectful and abusive care. One strength of our study is that because we surveyed all women who were on the maternity ward during the study period, we captured a range of experiences. Another strength is that we had the cooperation of hospital staff and administrators, who allowed us direct access to detained patients and who also agreed to be interviewed about their views of detention.

#### What is to be done

Many of the solutions key informants proposed were aimed at changing systems of insurance and payment. They did not frame the issue of detention in terms of human rights, although this is one lens through which others have viewed detention [2, 72].

In fact, we and others have argued that the practice of detention is a deviation from human rights standards, and is driven in part by inadequate enforcement of these rights in terms of national legislation and policies and local governance and leadership – specifically, of “the right to liberty, autonomy, self-determination, and freedom from coercion” asserted in Article VII of the Universal Charter on Respectful Maternity Care [37]. This right is upheld by at least five international standards, as noted above in the Background. National law in the DRC asserts a right to liberty: Article 67 of the Congolese penal code prohibits arbitrary detention [73]. Furthermore, detention violates the Convention on the Rights of the Child [38], of which the DRC is a signatory. The Convention clearly applies to the infants who are detained, but also, in some cases, to their mothers, given that the lower limit of age in our sample was sixteen.

We also argue that because post-partum detention is a deprivation of liberty that disproportionately penalizes women, it constitutes gender-based violence. Women have higher needs for health care than men because of their reproductive role, but frequently have fewer means to pay for that care [74], making them more likely to be detained for non-payment. As defined by CEDAW, gender-based violence is “violence that is directed against a woman because she is a woman or that affects women disproportionately” and includes “acts that inflict physical, mental or sexual harm of suffering, threats of such acts, coercion, and other deprivations of liberty.” [75]

In addition, detention especially penalizes those women who are most biologically, socially, and financially vulnerable [76]. An example of heightened biological vulnerability is that detained women in this sample were more likely to have had obstetric complications which in turn required costly interventions, including caesarean section, a common source of catastrophic costs [77]. In terms of social vulnerability, they were more likely to be single than women who were discharged as expected, and thus lacking in the emotional support of a partner and in-laws, as well as financial support to contribute to paying their fees. In terms of financial vulnerability, women who were detained for non-payment reported, on average, even lower incomes and less household wealth than the sample overall.

The Global Strategy for Women’s, Children’s and Adolescents’ Health provides a helpful framework to expand the discussion and address financial- and human rights-based drivers of detention at all levels in the DRC [1]. It envisions “[b] y 2030, a world in which every woman, child and adolescent in every setting realizes their rights to physical and mental health and well-being, has social and economic opportunities, and is able to participate fully in shaping sustainable and prosperous societies,” and centers human rights and gender equality in its approach.

Elements of the Global Strategy that are relevant to hospital detention of mothers and their infants for non-payment of fees include its calls for country leadership, increased government spending on health, reduced out-of-pocket expenditures, creation of national health insurance schemes, “equip [ping] the health workforce to provide good-quality, non-discriminatory care,” ensuring universal health coverage, providing financial protection for individuals and households to prevent catastrophic out-of-pocket health expenditures, and “strengthen [ing] legal frameworks to register and address human rights violations, promote human rights literacy and provide age- and gender-appropriate protection services and safe spaces for women, children and adolescents.” [1] DRC is a member of the Global Financing Facility to implement the Global Strategy, but the province of Haut-Katanga is not one of the fourteen provinces included in the initial stages [78].

## Conclusion

Over half of women delivering at this facility in Lubumbashi, DRC were detained without medical indication because they were not able to pay for care received. In addition, over one quarter of the infants born to these women had died during delivery or in the first three days of life. This represents a violation of human rights and a systemic failure to ensure that all people have access to essential health services and that they not suffer financial hardship due to the cost of those services, goals set forth by WHO member states in their commitment to work toward universal health coverage [79]. The Global Strategy for Women’s, Children’s and Adolescents’ Health [1] provides a roadmap to address the complex financial, human rights, and quality-of-care issues raised by this study.

## Data Availability

The datasets used and/or analysed during the current study are available from the corresponding author on reasonable request.

## Abbreviations

CDF: Congolese francs
CEDAW: Committee on the elimination of discrimination against women
DEVAW: Declaration on the elimination of violence against women
DRC: Democratic Republic of the Congo
ICCPR: International Covenant on Civil and Political Rights
ICESCR: International Covenant on Economic, Social and Cultural Rights
USD: United States dollars
WHO: World Health Organization

## Footnotes

1 Katanga was a single province until its partition into the provinces of Haut-Katanga, Tanganyika, Lualaba, and Haut-Lomami [https://en.wikipedia.org/wiki/Provinces_of_the_Democratic_Republic_of_the_Congo, accessed 18 Jun 18]
